# Microplastics influence on herbicides removal and biosurfactants production by a *Bacillus* sp. strain active against *Fusarium culmorum*

**DOI:** 10.1038/s41598-023-41210-5

**Published:** 2023-09-05

**Authors:** Aleksandra Walaszczyk, Anna Jasińska, Przemysław Bernat, Grażyna Płaza, Katarzyna Paraszkiewicz

**Affiliations:** 1 Doctoral School of Exact and Natural Sciences, Department of Industrial Microbiology and Biotechnology, Faculty of Biology and Environmental Protection, University of Lodz, Lodz, Poland; 2 Department of Industrial Microbiology and Biotechnology, Faculty of Biology and Environmental Protection, University of Lodz, Lodz, Poland; 3 Faculty of Organization and Management, Silesian University of Technology, Zabrze, Poland

**Keywords:** Environmental microbiology, Applied microbiology

## Abstract

The amounts of anthropogenic pollutants, e.g., microplastics (MPs) and pesticides, in terrestrial and aquatic ecosystems have been increasing. The aim of this study was to assess the influence of MPs on the removal of herbicides (metolachlor, MET; 2,4-dichlorophenoxyacetic acid, 2,4-D) and the production of biosurfactants (surfactin and iturin) by *Bacillus* sp. Kol L6 active against *Fusarium culmorum*. The results showed that Kol L6 eliminated 40–55% MET and 2,4-D from liquid cultures, but this process was inhibited in the presence of MPs. Although the pollutants did not strongly limit the production of surfactin, iturin secretion was found to decrease by more than 70% in the presence of all three pollutants. Interestingly, the strongest modification in the profile of iturin homologues was calculated for the cultures containing MET + MP and 2,4-D + MET + MP. The bacteria significantly limited the growth of the phytopathogenic *F. culmorum* DSM1094F in the presence of individual pollutants and their two-component mixtures. However, in the presence of all three tested pollutants, the growth of the fungus was limited only partially (by no more than 40%). The presented results are a starting point for further research on bacteria-fungi-plants interactions in the soil environment in the presence of multiple pollutants.

## Introduction

Chemical pesticides are produced synthetically and applied as a main method for pest removal, especially in agriculture. In 2020, pesticide consumption was 2.66 million metric tons, with the United States being the largest pesticide-consuming country worldwide with 407.8 thousand metric tons of pesticides used, and Brazil coming in second with 377.2 thousand tons consumed. From 1990 to 2010, the global consumption of pesticides increased by more than 50%^1^. According to Soloneski et al.^2^, more than 99.9% of pesticides applied to crops worldwide become toxic residues in the environment, never reaching their specific targets. These compounds are usually toxic and persist in both terrestrial and aquatic ecosystems^3^. The usage of pesticides results in their accumulation in the environment and, subsequently, a modification of the metabolic activity of various organisms and interactions between them^4^. The residues of pesticides can affect not only environmental biodiversity but also human health^3,5^. Among the various techniques used for pesticide removal, microbial bioremediation stands out as an eco-friendly, cost-effective and efficient method. Some microorganisms, primarily bacteria^6^ and fungi^7^, have been proven to be able to mineralize or transform (partially degrade and/or detoxicate) anthropogenic pollutants, including synthetic pesticides^3,8^.

2,4-dichlorophenoxyacetic acid (2,4-D), an herbicide containing phenoxyacetic acid), has been widely used for decades to control broadleaf weeds in several crops, including rice, wheat, sorghum, sugar cane, and corn^9^. According to Islam et al.^10^, over 1500 commercial herbicide products contain 2,4-D as an active ingredient. This herbicide has often been detected in surface and ground water and therefore poses an important environmental issue and health hazard. Importantly, approximately 91.7% of 2,4-D ends up in water. Among the phenoxyacetic acid herbicides, 2,4-D is one of the most easily broken-down compounds, and it has been discovered that microbial communities worldwide have a high prevalence of 2,4-D mineralization^11^.

Another widely used herbicide is MET (2-chloro-N-(2-ethyl-6-methylphenyl)-N-((1S)-2-methoxy1-methylethyl) acetamide), which accounts for approximately 4.2% of herbicide usage worldwide. This compound is a commonly used, highly selective chloroacetamide pesticide. MET has a moderate level of toxicity, an ability to block the enzymes involved in the gibberellin pathway and can exert negative effects on human liver cells^12^.

Environmental pollution arising from plastic waste is a major global concern and one of the greatest threats to global aquatic and terrestrial biodiversity. Plastic pollution has been recognized as one of the most critical challenges of modern societies worldwide^13–16^. Plastic can be categorized based on several different qualities, e.g., chemical composition, particle size, application possibilities, and physicochemical properties. MPs are defined as small particles of synthetic organic polymers with a diameter between 1 μm and 5 mm^11^. According to Nizzetto et al.^17^, up to 700,000 tons of MP is input into agricultural land in Europe and North America annually. Weithmann et al.^18^ estimated that 35 billion to 2.2 trillion pieces of MP enter cropped soils via compost application in Germany annually. Based on their origin, MPs can be divided into two categories: primary (manufactured intentionally and used especially as an ingredient of various personal care products) and secondary (generated through the fragmentation of plastic litter).

Because of its hydrophobic properties, large molecular weight, and lack of functional groups that microbes can identify, polyethylene (the most popular petroleum-based plastic) persists in the environment^19^. Low-density polyethylene (LDPE) is difficult to degrade after disposal, which results in environmental pollution and ecosystem disruption. This type of MP also poses an increasing threat to terrestrial and aqueous wildlife. According to Sen and Rout^20^, there are many reports suggesting that polyethylene causes blockages in the intestines of birds and various aqueous animals.

Bacteria from the genus *Bacillus* are gram-positive, spore-producing rod-shaped, aerobes or facultative aerobes, mostly isolated from soil. According to Caulier et al.^21^, more than 370 *Bacillus* species have been described thus far. Members of the genus *Bacillus* are known to secrete a wide variety of metabolites, including enzymes, cyclic lipopeptide (cLP) biosurfactants belonging to the surfactin, iturin or fengycin families, polyketides, hybrids of polyketides and nonribosomal compounds and volatile compounds^21,22^. *Bacillus* cLPs are synthesized via nonribosomal peptide synthetases, which causes a significant amount of structural variability in these compounds, resulting in the production of a mixture of homologues (varying in the length of the fatty acid chain) and/or isoforms (differing in the amino acid sequence of the peptide)^23^. Many published data indicate that environmental *Bacillus* strains usually secrete biosurfactants as mixtures of various isomers and/or homologues. For example, while testing 25 *Bacillus* strains isolated from vegetable rhizospheres, Bartal et al.^24^ distinguished as many as 158 surfactin variants present in the ferment broths. The percentage content of different isoforms and homologues of *Bacillus* cLP biosurfactants can be affected by various factors, such as culture conditions and medium composition^25,26^.

Some of these metabolites produced by rhizospheric *Bacillus* bacteria exhibit antimicrobial properties, act as surfactants or play a role in improving plant health and growth; therefore, many of those species are described as plant growth-promoting rhizobacteria (PGPR). The mechanisms used by rhizobacteria (including PGPR) for the bioremediation of contaminated soils include biosorption/degradation/transformation of pollutants and production of various metabolites, e.g., polymeric substances, siderophores or biosurfactants^27^. Biosurfactant-producing *Bacillus* strains are one of the most promising PGPR, among others, due to the synergistic effect of surfactin, iturin and fengycin on bacterial motility leading to biofilm formation and root colonization, induced systemic resistance (ISR) in plants and antimicrobial activity against phytopathogens. In particular, bacterial biofilm presence as well as iturin and fengycin secretion play a significant role in plant protection against phytopathogens^21,28–30^. Some *Bacillus* strains are known as microorganisms capable of synthetic pesticide degradation^31^.

Anthropogenic pollutants such as herbicides and plastics may influence the diversity and/or metabolic activity of soil microbiota, including PGPR. The objective of this study was to evaluate whether the presence of MPs influences the ability of *Bacillus* bacteria to remove herbicides (2,4-D and MET), produce biosurfactants (surfactin and iturin) and inhibit fungal growth. The present study provides new data on the influence of MPs and herbicides on *Bacillus* sp. The ability of Kol L6 to produce biosurfactants and degrade herbicides allows for a better understanding of the impact of some pollutants on plant growth-promoting microorganisms. Based on our literature survey, this study is the first to report on the influence of MPs on 2,4-D and MET removal as well as biosurfactant production by a *Bacillus* strain active against phytopathogenic fungi from the genus *Fusarium*.

## Materials and methods

### Microbial strains

All the bacterial strains used in the study are stored in the strain collection of the Department of Industrial Microbiology and Biotechnology, University of Lodz, Poland. The *Fusarium culmorum* DSM 1094F strain was obtained from the Leibniz Institute DSMZ.

In this study, 18 different *Bacillus* strains isolated from rhizosphere with potentially different kinds and levels of anthropogenic pollutants were used (Table 1). Two of the strains, I’-1a and Zg 1.3, were isolated from highly polluted environments. The remaining 16 strains were isolated from nonurban areas. All of the strains secreted compounds capable of surface tension reduction (indicating their ability to produce biosurfactants), which was assessed by methods described in Materials and Methods, subsection “Biosurfactant extraction and analysis”.

**Table 1:** Isolation site of bacterial strains and biosurfactants production by the strains cultured for 72 h in LB medium. Surface activity was assessed by the DCT.

Bacterial strain	Site of soil sample isolation	DCT (mm)*	Biosurfactants production mg L^−1^ **		
			Surfactin	Iturin	Fengycin
I′-1a	Oil rafinery, Czechowice-Dziedzice, Poland (49°5′N 19°0′E)	7.5	6.7 ± 0.5	61.8 ± 4.1	+
Zg 1.3	Landfill of the former “Boruta” Dye Industry Plant in Zgierz, Poland (51°8′N 19°3′E)	8.5	70.2 ± 3.3	133.7 ± 9.36	+
IM 14	Helenów Public Park in Łódź, Poland (51°4′N 19°2′E)	7.0	15.8 ± 1.5	9.1 ± 0.5	+
KAS 1.10	Kashubian Landscape Park, Poland (54°1′N 18°3′E)	5.0	0.0 ± 0.0	27.7 ± 2.9	−
KAS 2.2		9.0	42.7 ± 3.0	255.9 ± 15.7	−
KAS 2.4		10.0	38.8 ± 3.1	283.5 ± 17.5	+
KAS 2.5		9.5	33.8 ± 2.3	23.1 ± 1.6	+
KAS 4.1		5.0	0.7 ± 0.1	10.5 ± 0.7	+
Kol B1	Kolnica village, Greater Poland Voivodeship (52°5′N 18°3′E)	8.0	101.2 ± 7.1	243.3 ± 16.9	−
Kol B9		9.0	33.5 ± 2.3	199.1 ± 13.9	+
Kol D3		9.0	67.3 ± 6.8	159.5 ± 15.8	+
Kol L2		5.0	14.0 ± 1.4	0.0 ± 0.0	+
Kol L6		8.5	38.4 ± 2.7	161.3 ± 11.3	+
Kol S3		10.0	7.9 ± 0.6	47.3 ± 4.8	+
Kol S5		6.0	21.2 ± 2.1	0.0 ± 0.0	−
Kol S8		9.5	27.2 ± 2.8	162.9 ± 11.4	+
Kol S10		4.5	0.0 ± 0.0	29.8 ± 1.4	−
Kol Si6		8.0	27.4 ± 1.9	368.3 ± 27.8	−

*Surface activity assessed by DCT for the negative control (LB medium) was 3 mm and for the positive control (5% water solution of SDS) it was 8 mm. The average standard deviation was lower than 10%, and therefore was not shown.

**Surfactin and iturin were evaluated by LC–MS/MS using appropriate standards and fengycin presence was assessed by MALDI-TOF/TOF.

### Chemicals

Chemicals used for surfactant and herbicide extraction by the QuEChERS method, surfactin and iturin standards, herbicides (2,4-D and MET), crystal violet solution for microscopic analysis and sodium dodecyl sulfate (SDS) were obtained from Sigma‒Aldrich (Germany). A stock solution of each herbicide (10 mg mL^−1^) was prepared in 96% ethanol obtained from Avantor Performance Materials Poland S.A. (Poland). Chemicals used in the analysis performed by liquid chromatography tandem mass spectrometry (LC–MS/MS) were purchased from Avantor Performance Materials Poland S.A. (Poland).

### MP preparation

In this study, LDPE powder with a grain size of 100–500 μm and a melt index of 190 °C/2.16 kg (Abifor AG, Switzerland) was used. Nonsterile LDPE (1.5 g) was suspended in 5 mL of 96% ethanol and incubated for an hour. During the incubation, the sample was vortexed several times. Then, 10 mL of sterile deionized water was added to obtain a final MP concentration of 100 mg mL^−1^. The procedure was performed under sterile conditions. The MP stock solution was subsequently used for the preparation of submerged bacterial cultures.

### Submerged bacterial cultures

Liquid Luria–Bertani (LB) medium was inoculated with a 1% 24-h-old preculture grown in the same medium, according to the method described by Płaza et al.^32^. Cultivation variants with the addition of 1) MP; 2) 2,4-D; 3) MET; 4) MP and 2,4-D; 5) MP and MET; and 6) MP, 2,4-D and MET were prepared. The final concentration of MP in the culture was 2000 mg L^−1^, while the final concentration of each herbicide was 50 mg L^−1^. To determine the antifungal activity, analogous cultures with the addition of *F. culmorum* DSM 1094F (using a 10% 24-h-old fungal preculture obtained in LB medium) were prepared. Appropriate biotic controls (bacterial or fungal culture with neither MP nor herbicide addition) and abiotic controls (with no microorganism addition) were also prepared. The results of the preliminary study confirmed that the amount of ethanol used for herbicide and MP stock preparation does not have any effect on bacteria and fungi. The cultures in the final volume of 30 mL were incubated for 72 h in 100 mL Erlenmeyer flasks. All cultures and abiotic controls were incubated on a rotary shaker (120 rpm) at 28 °C. Then, in the experiments on antifungal activity, the fungal biomass was separated from the extracellular fluid by vacuum filtration using previously weighed Whatman filter paper No. 1 (Merck, Germany). The mycelium was then dried at 60 °C to reach a constant weight.

### Herbicide extraction and analysis

First, to assess the influence of MPs on herbicide recovery, abiotic controls were prepared. The data of abiotic control individual variants were assumed to be 100% to determine the level of pesticides in the appropriate cultures containing *Bacillus* sp. Kol L6 bacteria. Then, MP (2 g L^−1^) was added to LB medium containing MET and/or 2,4-D (50 mg L^−1^) and incubated for 72 h on a rotary shaker, followed by extraction and analysis of individual herbicide contents. Finally, 2,4-D and MET removal by *Bacillus* sp. Kol L6 cultured in the presence of individual pollutants or their mixtures was assessed.

To estimate herbicide elimination by the bacteria, a sample of the bacterial culture (20 mL) was homogenized twice with glass beads (1 mm diameter) for 4 min at 25 m s^−1^ (Retsch, Ball Mill MM 400). To the obtained homogenized cultures, 10 mL of acetonitrile (ACN) and four salts (2 g of MgSO_4_, 0.5 g of NaCl, 0.5 g of C_6_H_5_NaO_7_ × 2H_2_O, and 0.25 g of C_6_H_6_Na_2_O_7_ × 1.5 H_2_O) were added, and the extraction was carried out using QuEChERS techniques. After centrifugation, the top layer was collected. 2,4-D and MET contents were analysed according to the methods described by Nykiel et al.^33^ and Jasińska et al.^34^, respectively. Briefly, 2,4-D content was measured using an Agilent 1200 HPLC system (Agilent, USA) and a 3200 Q-TRAP mass spectrometer (Sciex, USA) equipped with an ESI source. The obtained extract was diluted in a mixture of water and methanol (80:20, *v*/*v*). The parameters of the chromatographic method were as follows: 10 μL of the diluted sample was injected onto a Kinetex C18 column (50 mm × 2.1 mm, particle size: 5 μm; Phenomenex, Torrance, CA, USA) and heated at 37 °C with a flow rate of 500 μL min^−1^. Water (A) and methanol (B) were applied as mobile phases, with both containing 5 mM ammonium formate. The solvent gradient was initiated at 80% and, after 1 min, decreased to 10% A for 1 min; then, it was maintained at 10% A for 2.5 min before returning to the initial solvent composition over 2 min. The ion source of MS was operated in negative mode at a temperature of 500 °C. The multiple reaction monitoring pairs (MRM) for 2,4-D were m/z 219.9–160.9 and 220.9–162.9. The same chromatographic parameters were applied for MET. However, the MS parameters were different. The ESI source was operated in positive mode, where the MRM pairs for MET were m/z 284.09–252.2 and 284.09–176.3.

### Biosurfactant extraction and analysis

In a preliminary study, the drop collapse test (DCT) was used as a sensitive, rapid method for screening bacteria producing biosurfactants^35,36^. Briefly, the culture was centrifuged, and 10 μL of the supernatant was placed on the surface of a polystyrene plate. After 15 min, the diameter of the drop was measured and compared to the positive and negative control drop diameters (5% water solution of SDS and LB medium, respectively).

To determine the production of cLPs, the culture was centrifuged at 10,000×*g* for 10 min. The supernatant was extracted by a modified QuEChERS technique according to the method previously described by Bernat et al.^37^ and Paraszkiewicz et al.^25,38^. Briefly, the process included six steps: (1) a 5 mL sample of the culture supernatant was mixed rapidly with 5 mL of distilled water in a 50 mL centrifuge tube; (2) 10 mL of ACN was added, and the sample was vortexed for 2 min; (3) the mixture of the four salts (2 g of MgSO_4_, 0.5 g of NaCl, 0.5 g of C_6_H_5_NaO_7_ × 2H_2_O, and 0.25 g of C_6_H_6_Na_2_O_7_ × 1.5 H_2_O) was added, and the sample was vortexed for five minutes; (4) the sample was left to sit for 20 min; (5) the upper organic phase was pooled; (6) another 10 mL portion of ACN was introduced to the sample, and the extraction procedure was repeated.

Surfactin and iturin analyses were performed using an Agilent 1200 LC (Santa Clara CA, USA) system with a 3200 QTRAP mass spectrometer (Sciex, Framingham, MA, USA) equipped with an ESI source. Five microliters of ACN samples was injected onto a Kinetex C18 column (50 mm × 2.1 mm, particle size: 5 μm; Phenomenex, Torrance, CA, USA) heated to 40 °C with a flow rate of 600 μL min^−1^. The mobile phase consisted of water (A) and methanol (B), which were both supplemented with 5 mM ammonium formate. The run time was 6 min with the solvent gradient was initiated at 40% B. After 0.5 min, the amount of B was increased to 100% during the next 1.5 min and was maintained at 100% for 2.5 min before returning to the initial solvent composition over the next 2 min. The mass spectrometer ion source worked in positive mode, spray voltage 5.500 V and temperature 600 °C. The data analysis was performed with Analyst™ v1.5.3 software (Sciex, Framingham, MA, USA). Quantitative lipopeptides analyses were performed for surfactin and iturin A standards (Merck). The monitored multiple reaction monitoring (MRM) pairs were m/z 1030–391, 1044–391, 1058–391 and 1072–391 for sodiated molecules [M + Na]^+^ of the surfactin homologues C13, C14, C15 and C16, respectively. For sodiated ions of homologues C13, C14, C15 and C16 of iturin A, the MRM pairs were m/z 1051.5/1051, 1065.5/1065, 1079.5/1079 and 1093.5/1093, respectively.

Fengycin presence was confirmed using a MALDI-time of flight (TOF/TOF) mass spectrometer AB SCIEX 5800 System (AB Sciex)^38^. Briefly, a mixture of a 0.5 μL of cLP extract (diluted in 2 mL of methanol) and 0.5 μL of a matrix solution (containing 10 mg mL^−1^ DHB dissolved in ACN) was deposited onto the MALDI target. MALDI-TOF/TOF analyses were conducted in the positive ionization and reflector mode in the range m/z 900–2000 at a fixed laser intensity of 3500 (instrument-specific units). The ten most intense signals per spot were selected for automated MS/MS measurement at a fixed laser intensity of 5000 (instrument-specific units).

### Microscopy

Microscopic analysis was performed using a Nikon Eclipse 50i light microscope with a Nikon DS-Fi3 camera and NIS-Elements D 5.11.01 software.

### Statistical analysis

All experiments were conducted at least in triplicate. The obtained results were presented as their mean values. An average standard deviation (SD) was calculated. One-way ANOVA analysis of variance was performed using Microsoft® Excel® (Microsoft Corporation, Washington, DC, USA). Differences at *p* ≤ 0.05 were considered significant.

## Results and discussion

### Characterization of biosurfactant production by the bacterial strains

The results obtained from the DCT in the preliminary study revealed that all tested strains secreted compounds with surface activity. Further analysis using LC‒MS/MS and MALDI-TOF/TOF techniques showed that all tested strains produced cLPs. Moreover, these strains exhibited diverse profiles of surfactin, iturin and fengycin secretion (Table 1). Exemplary LC‒MS/MS chromatograms (Figure S1) and MALDI-TOF/TOF mass spectrometry (MS) spectra (Figure S2) of the lipopeptides extracted from *Bacillus* sp. Kol L6 cultures are presented in the supplementary material.

Most of the strains (11 isolates) simultaneously produced surfactin, iturin and fengycin. In turn, a mixture of two biosurfactants (surfactin and iturin) was produced by three strains, and the secretion of only one type of biosurfactant (surfactin or iturin) was found in four strains. Depending on the strain, the levels of secreted surfactin and iturin varied and were in the ranges of 0.7 ± 0.1 to 101.2 ± 7.1 mg L^−1^ and 9.1 ± 0.5 to 368.3 ± 27.8 mg L^−1^, respectively.

### Comparison of the ability of the *Bacillus* strains to remove herbicides

**Figure 1:**
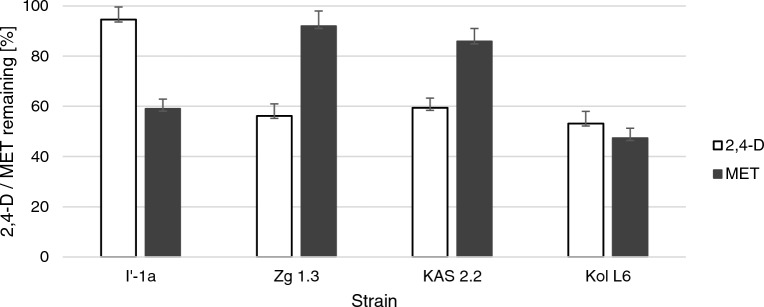
Percentage values of 2,4-D and MET (50 mg L^−1^) remaining in the cultures of selected *Bacillus* strains cultivated for 72 h in LB medium on a rotary shaker (120 rpm) at 28 °C. Percentage values of the pesticide remaining in abiotic control was taken as 100%. The significance of the differences between the samples was determined according to ANOVA test (*p* ≤ 0.05).

Of the 18 studied strains (Table 1), only four isolates showed a notable (> 40%) ability to remove at least one of the tested pesticides from the growth environment (Fig. 1). The most effective was *Bacillus* sp. Kol L6. This strain removed 2,4-D and MET with 53.2 and 47.4% efficiency, respectively; therefore, it was chosen for further analysis.

Pesticide residues present in the soil adversely affect the populations of organisms inhabiting it. They can both disrupt the functioning of individual organisms and affect the interactions between them^39–41^. The wide use of 2,4-D in agriculture has led to the long-term persistence of its residues in agricultural soils. Many scientific works prove its adverse effect on plants and soil organisms such as earthworms, fungi and bacteria and their interrelationships^42^.

There are only a few papers showing the degradation of 2,4-D by bacteria of the genus *Bacillus*. Karami et al.^43^ isolated bacteria from polluted wheat field soils and proved their capacity to degrade 2,4-D. The identified strains were classified as members of the genera *Pseudomonas*, *Entrobacter*, *Bacillus*, *Seratia* and *Staphylococcus*. Similarly, Nguyen et al.^44^ demonstrated efficient degradation of 2,4-D by a population of microorganisms present in heavily contaminated soil. In addition, prolonged exposure to the herbicide resulted in the enrichment of the community in bacteria of the genera *Bacillus* and *Paenibacillus*, which may suggest an increased predisposition of these genera to tolerate and degrade 2,4-D.

To date, many MET-degrading microorganisms, including bacterial and fungal strains, have been identified^12,45–47^. Some studies have reported the ability of *Bacillus* bacteria to mineralize or transform MET^48,49^. Kanissery et al.^50^ used DNA-stable isotope probing to investigate microbial MET mineralization in soil. The 16S rRNA gene sequencing of aerobic MET-mineralizing microorganisms showed a close relationship to *Bacillus* spp. Nevertheless, those reports did not evaluate the ability of bacillus strains to produce biosurfactants.

Importantly, the strain chosen for further analysis in this study (*Bacillus* sp. Kol L6) was established as a very effective producer of both surfactin (38.4 ± 2.7 mg L^−1^) and iturin (161.3 ± 11.3 mg L^−1^) (Table 1). Similar levels of surfactin (35.0 ± 2.6 mg L^−1^) and iturin (132.5 ± 15.6 mg L^−1^) secretion were reported for the natural surfactin and iturin overproducer *B. subtilis* IM 13^38^. To the best of our knowledge, there have been no reports on biosurfactant-producing *Bacillus* strains capable of 2,4-D and/or MET removal.

### 2,4-D and MET removal by *Bacillus* sp. Kol L6 from cultures amended with a mixture of the herbicides and/or MP

**Figure 2:**
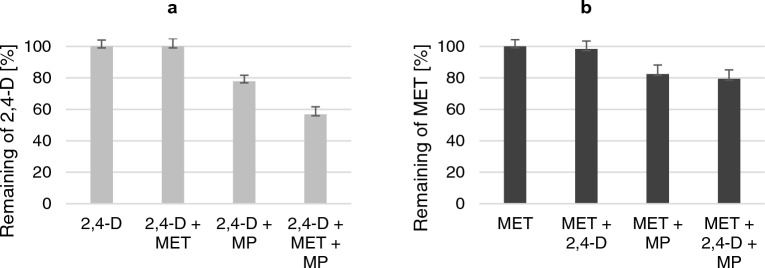
Abiotic controls: MP influence on the remaining percentage of (**a**) 2,4-D and (**b**) MET from LB medium after 72 h of incubation on a rotary shaker (120 rpm), at 28 °C. The significance of the differences between the samples was determined according to ANOVA test (*p* ≤ 0.05).

To assess MP influence on herbicide recovery, abiotic controls were prepared (Fig. 2).

It was found that in the systems containing 2,4-D or MET alone, close to 100% of the pesticides remained in the sample after incubation (Fig. 2). In the case of both 2,4-D and MET, the addition of the second herbicide used in the study did not affect the extraction efficiency. However, it was found that in the presence of MP, only approximately 80% of 2,4-D and MET remained in the sample after incubation. Moreover, in a mixture containing MET and MP, the remaining amount of 2,4-D was reduced by approximately 40%.

**Figure 3:**
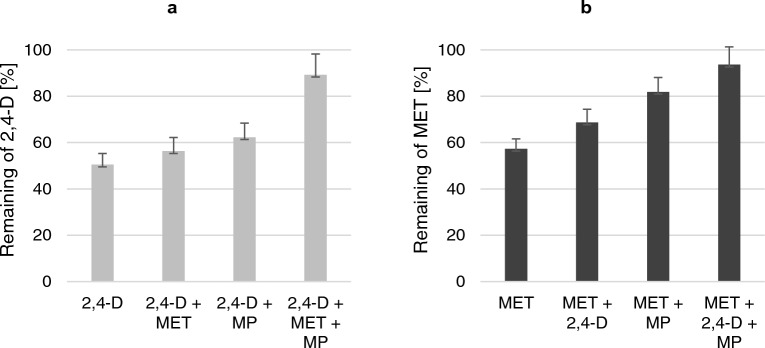
Influence of MP on the percentage of the remaining (**a**) 2,4-D and (**b**) MET from *Bacillus* sp. Kol L6 cultures conducted in LB medium after 72 h of cultivation on a rotary shaker (120 rpm), at 28 °C. The results are shown as a percentage of control samples (Fig. 2). The significance of the differences between the samples was determined according to ANOVA test (*p* ≤ 0.05).

As shown in Fig. 3, *Bacillus* sp. Kol L6 showed the ability to eliminate 2,4-D and MET from both the cultivations containing individual pollutants or their mixtures (containing more than one pollutant). Compared to the cultures containing a single pesticide, the removal of 2,4-D and MET in the presence of MP decreased by 11.8 and 24.6% (*p* ≤ 0.05), respectively. However, the addition of both herbicides as well as MP almost completely reduced the removal of both 2,4-D and MET. The effectiveness of 2,4-D and MET elimination from multicomponent systems was only 10.7 and 6.4%, respectively (p < 0.003).

Taking into account the previously demonstrated possibility of 2,4-D and MET sorption on the surface of MP particles, the reduced efficiency of eliminating these herbicides from the growth environment by *Bacillus* sp. Kol L6 may result from the limited bioavailability of the pesticides used. Because of the small MP particle size, large specific surface area and strong hydrophobicity, pesticides could be easily adsorbed onto MP surfaces^51,52^. Information about how MPs could impact microbial degradation activity towards pesticides has seldom been reported. However, there are papers describing the effect of MPs on prolonging the effective residual life of cypermethrin and modifications of some parameters of soil microbial activity in the presence of glyphosate^53,54^.

### Influence of herbicides and MP on surfactin and iturin production by *Bacillus* sp. Kol L6

**Figure 4:**
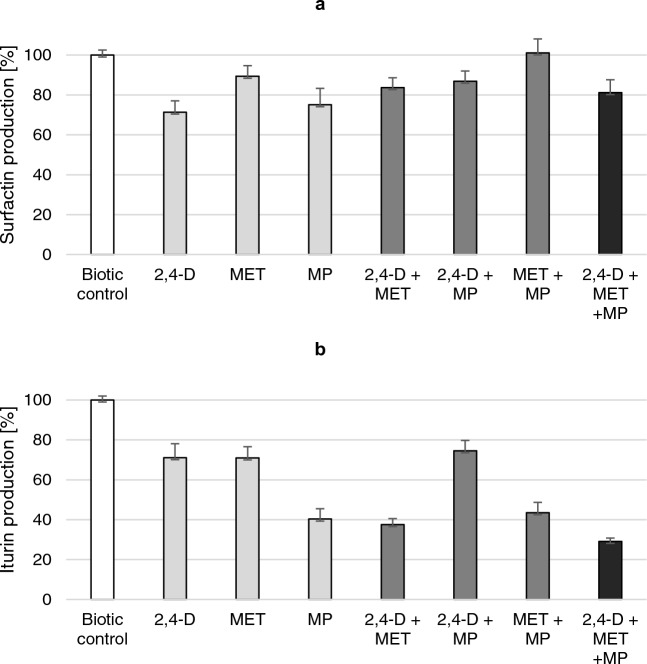
Surfactin (**a**) and iturin (**b**) production by *Bacillus* sp. Kol L6 in the cultivations containing individual pollutants: 2,4-D; MET or MP or pollutant mixtures. The results are shown as a percentage of biotic control data. Legend: white square—without pollutants, light grey square—addition of one pollutant, dark grey square—addition of two pollutants, black square—addition of three pollutants. The significance of the differences between the samples was determined according to ANOVA test (*p* ≤ 0.05).

Surfactin production in the presence of 2,4-D, MET and MPs (used alone or as pollutant mixtures) is shown in Fig. 4a. It was established that the pollutants do not strongly limit surfactin production, though the differences were found to be statistically significant (*p* < 0.001). The lowest level of surfactin was found in the culture containing 2,4-D or MP (71.4 ± 5.6 and 75.1 ± 8.2%, respectively) compared to 100% calculated for the biotic control (without any pollutant addition). Interestingly, MET added together with MP did not negatively affect surfactin synthesis.

In contrast, the use of each pollutant alone or pollutant mixtures resulted in the reduction of iturin production by *Bacillus* sp. Kol L6 (Fig. 4b) (*p* < 0.001). The lowest iturin production was noted for cultures containing a mixture of all pollutants (29.1 ± 1.7%), a 2,4-D + MET mixture (37.6 ± 3.0%) and MP added separately (40.3 ± 5.2%).

**Figure 5:**
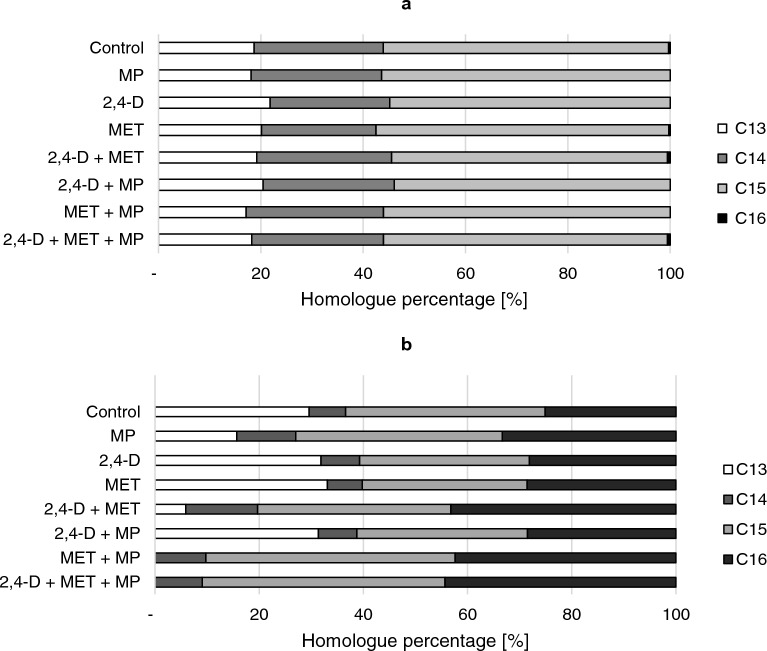
Surfactin (**a**) and iturin (**b**) homologues produced by *Bacillus* sp. Kol L6 in the presence of different pollutants: 2,4-D; MET or MP or their mixtures.

Four homologues of surfactin (C13-16) and four homologues of iturin (C13-16) were found in the control culture (Fig. 5). In all of the examined cultures, variant C15 of surfactin was dominant, ranging between 53.9 and 57.3%. The obtained results revealed that the pollutants used in the study did not significantly influence the surfactin homologue pattern. Exemplary chromatograms of the identified surfactin homologues are available in the supplementary material in Figure S3.

Interestingly, a complete reduction in the content of iturin homologue C13 was observed for cultures containing the MET + MP mixture and the mixture of all three pollutants. On the other hand, the relative contents of C15 and C16 homologues in the same samples visibly increased from 38.3 to 47% and from 25.1 to 43%, respectively.

The obtained data revealed that the simultaneous presence of an herbicide mixture and MP strongly disturbed *Bacillus* sp. Kol L6 iturin production and affected the structural diversity of iturin.

Many publications show that 2,4-D, MET and MP may influence the metabolic activity of microorganisms. For example, Bernat et al.^55^ showed that the fungal strain *Trichoderma harzianum* IM 0961 can reduce oxidative stress in 2,4-D-affected wheat and alleviate its toxic effect. Hogan et al. ^56^ showed that the tfdA gene, which encodes 2,4-D/a-ketoglutarate dioxygenase catalysing the first step of 2,4-D degradation, is often found in soil bacteria from the genera *Bacillus*, *Lactobacillus* and *Streptococcus*. Yang et al.^54^ showed that in soil polluted with the pesticide glyphosate as well as MP, microbial respiration and the dynamics of some soil enzymes (β-glucosidase, urease and phosphatase) changed significantly. However, there are no previous studies on the impact of MP on *Bacillus* surfactin and iturin production.

Importantly, the subtle structural differences in biosurfactant variants (produced as a mixture of various isoforms and/or homologues) might modify the biological activity of these compounds^57,58^. However, future research is necessary to evaluate the precise antimicrobial action of individual *Bacillus* surfactant homologues. In the presence of pollutants, the efficiency of cLP biosurfactant production by *Bacillus* strains may change. However, the effect of MPs, 2,4-D and MET on the biosynthesis of bacterial lipopeptides such as surfactin and iturin has not been reported thus far.

### Influence of herbicides and MPs on the antifungal activity of *Bacillus* sp. Kol L6

**Figure 6:**
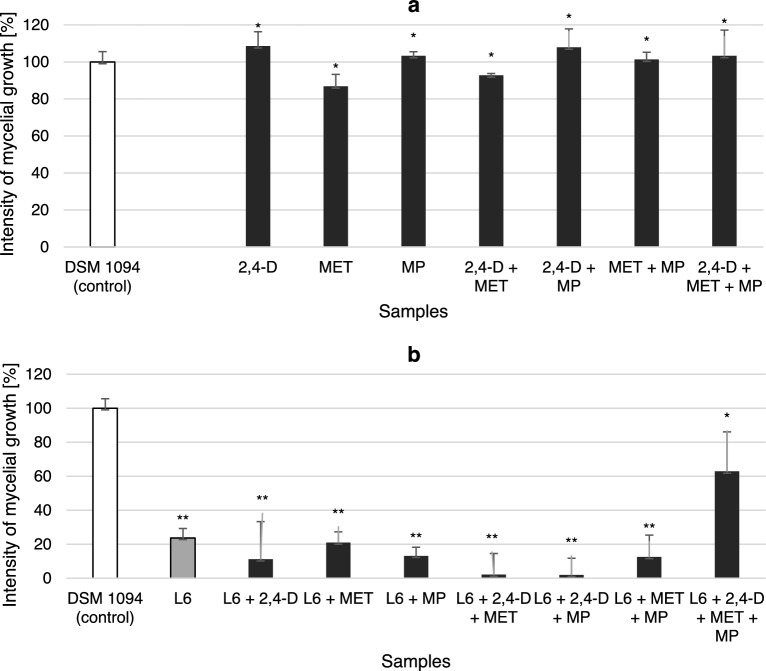
*F. culmorum* DSM 1094F growth intensity in LB medium amended with the pollutants: 2,4-D; MET or MP. Dry fungal biomass was obtained from (**a**) fungal cultures and (**b**) mixed bacterial-fungal cultures. The significance of the differences between the samples was determined according to ANOVA test (*p* ≤ 0.05). * no significant differences in fungal growth compared to control (*p* > 0.05); ** significant differences in fungal growth compared to control (*p* < 0.001).

To test the influence of the pollutants on *Bacillus* sp. Kol L6 antifungal activity and mycelial growth (Fig. 6a) were examined in the presence of pollutants and used as reference data in further analysis containing the addition of bacteria Kol L6 (Fig. 6b).

Pollutants (used individually or in mixtures) did not show a significant inhibitory effect on *F. culmorum *DSM 1094F growth (Fig. 6a) (*p* > 0.05). In contrast, in the mixed fungal-bacterial culture containing the fungus and *Bacillus* sp. Kol L6 bacteria, a very strong (nearly 80%) inhibitory effect was observed (Fig. 6b). Similarly, in the presence of individual pollutants and their mixtures, the bacteria also strongly inhibited the growth of the fungus (by more than 86.8%). The only exception was the culture containing all three pollutants (2,4-D, MET and MP), in which the growth of the fungus was limited only partially (by 37.2%). With the exception of the sample containing all three impurities, all other results showing a decrease in fungal growth were statistically significant, with a p-value lower than 0.001.The obtained results correspond with the data presented for iturin production obtained for *Bacillus* sp. Kol L6 cultured in LB medium amended with both herbicides and MPs.

Many papers emphasize the strong antifungal activity of iturin. For example, Calvo et al.^59^, using the *Bacillus amyloliquefaciens* BUZ-14 strain capable of producing surfactin, iturin and fengycin, showed that iturin A is the main lipopeptide family responsible for fungal inhibition. Xiao et al.^60^ examined the activity of fengycin and iturin A isolated from *B. subtilis* Z-14 on the soil-born fungus *Gaeumannomyces graminis* var. *tritici*. They established that iturin A induced cell wall disappearance, membrane degeneration, intracellular material shrinkage, and hyphal fragmentation, while fengycin destroyed the internal structure of fungal cells.

### Microscopic analysis

**Figure 7:**
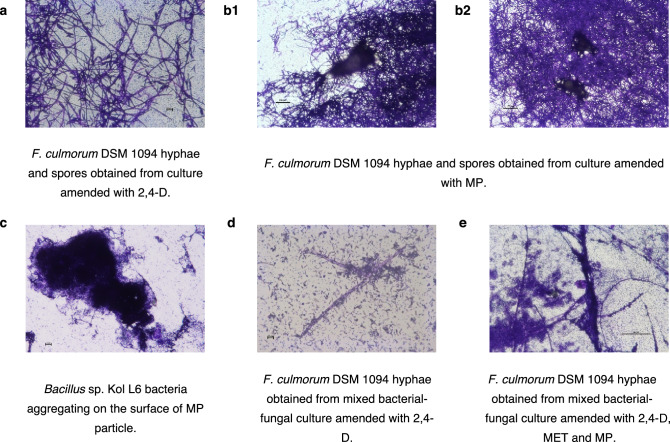
Microscopic analysis of selected samples withdrawn from mixed *F. culmorum* DSM 1094F and *Bacillus* sp. Kol L6 cultures amended with selected pollutants: 2,4-D; MET or MP.

Microscopic analysis showed that *F. culmorum *DSM 1094F cultivated in LB medium grew homogeneously and abundantly without pellet formation regardless of the presence of herbicides (Fig. 7a). The fungal hyphae formed loose aggregates around MP particles; however, no pellets were observed (Fig. 7b). Microscopic analysis revealed that *Bacillus* sp. Kol L6 had the ability to form biofilms on MPs (Fig. 7c). In the bacterial-fungal mixed culture, after 72 h of cultivation, a nearly complete reduction in fungal biomass occurred (Fig. 7d). The incubation of bacteria and the fungus in the same environment resulted in the formation of very short filaments with no branches. Moreover, it was observed that the majority of hyphal tips lost their integrity. When the mixed bacterial-fungal culture was amended with all of the pollutants used in the study, *Bacillus* sp. Kol L6 antifungal activity was limited, which resulted in moderate fungal growth (Fig. 7e). These results correspond well with the data presented in Figs. 4b and 6b (presenting iturin production and fungal biomass reduction, respectively).

Presently, many scientific papers have focused on the ability of various microorganisms to degrade MPs^61–64^. However, the influence of MPs on microorganism metabolic activity (e.g., herbicide biodegradation or biosurfactant production) is rarely researched. One such paper was that of Jasińska et al.^34^, who reported that MPs induced oxidative stress in the MET-degrading filamentous fungus *T. harzianum*.

The results obtained in this study suggest that Kol L6 bacteria have the ability to form biofilms on the surface of MP particles. Similarly, the study conducted by Tarafdar et al.^65^ revealed that *Bacillus siamensis* ATKU1 formed biofilms on the MP surface after its interaction with LDPE as a sole carbon source.

## Conclusion

The present work is the first to show the influence of MPs on the degradation of selected herbicides, the combined effect of these pollutants on the production of surfactin and iturin by *Bacillus* bacteria and their antifungal activity. It has been shown that *Bacillus* sp. Kol L6 is capable of eliminating 2,4-D and MET, but in the presence of plastic microparticles, this process is strongly disturbed. In addition, both herbicides and MPs had little effect on the level of surfactin produced but strongly limited the biosynthesis of iturin. This was especially noticeable in cultures containing a mixture of 2,4-D + MET or 2,4-D + MET + MPs. In addition, one of the iturin homologues was completely absent under these conditions. *Bacillus* sp. Kol L6 strongly limited the growth of the phytopathogenic fungus *F. culmorum* DSM 1094F. Moreover, the addition of a mixture of 2,4-D and MET and a mixture of 2,4-D and MP increased the antifungal activity of the bacteria. However, in cultures containing all three contaminants, fungal growth was limited by only 40%. It can therefore be concluded that in the presence of MPs and the mixture of tested herbicides, the bacteria show a weaker antifungal effect. Considering the common co-occurrence of MPs and other pollutants in the natural environment, this paper introduces new information that may be a starting point for further research, e.g., on bacterial-fungus‒plant interactions in the soil environment and the influence of soil pollutants on the metabolic activity of plant growth-promoting (PGP) microorganisms.

### Supplementary Information

Supplementary Information.

## Data Availability

The data presented in this study are available on request from the corresponding author.
