# Supplementary material for: Microplastics influence on herbicides removal and biosurfactants production by a Bacillus sp. strain active against Fusarium culmorum
Source: Sci Rep. 2023 Sep 5;13:14618. doi: 10.1038/s41598-023-41210-5 (PMC10480202; doi:10.1038/s41598-023-41210-5)
Supplement: Supplementary file 1 — Supplementary Information. [file 41598_2023_41210_MOESM1_ESM.pdf]

a

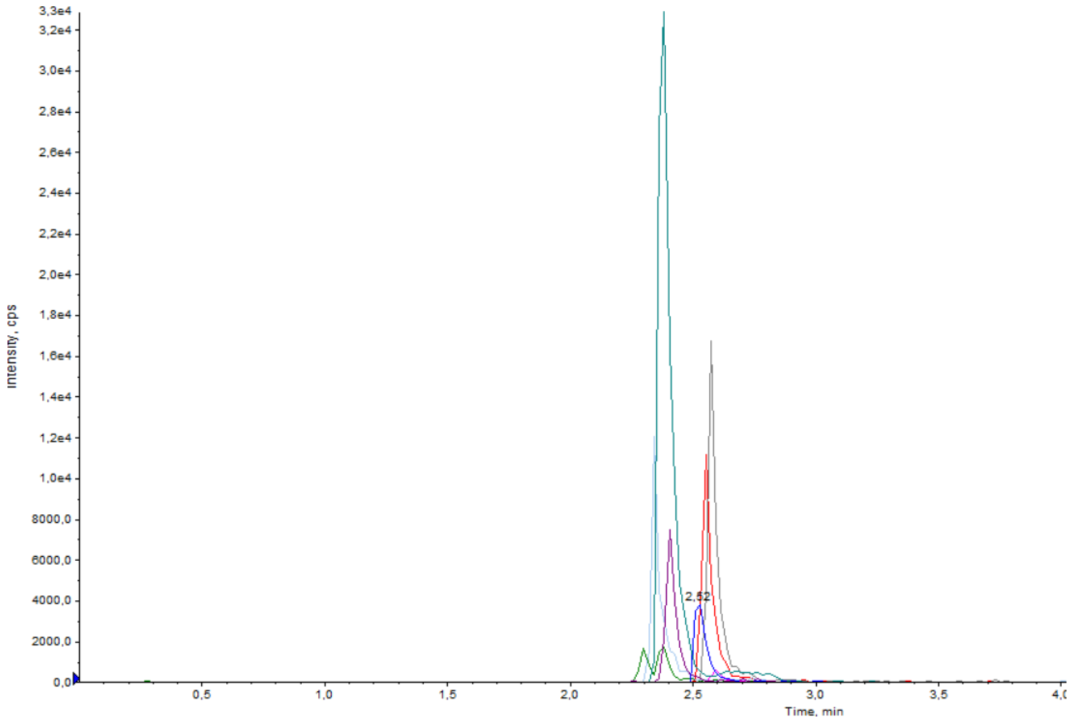

b

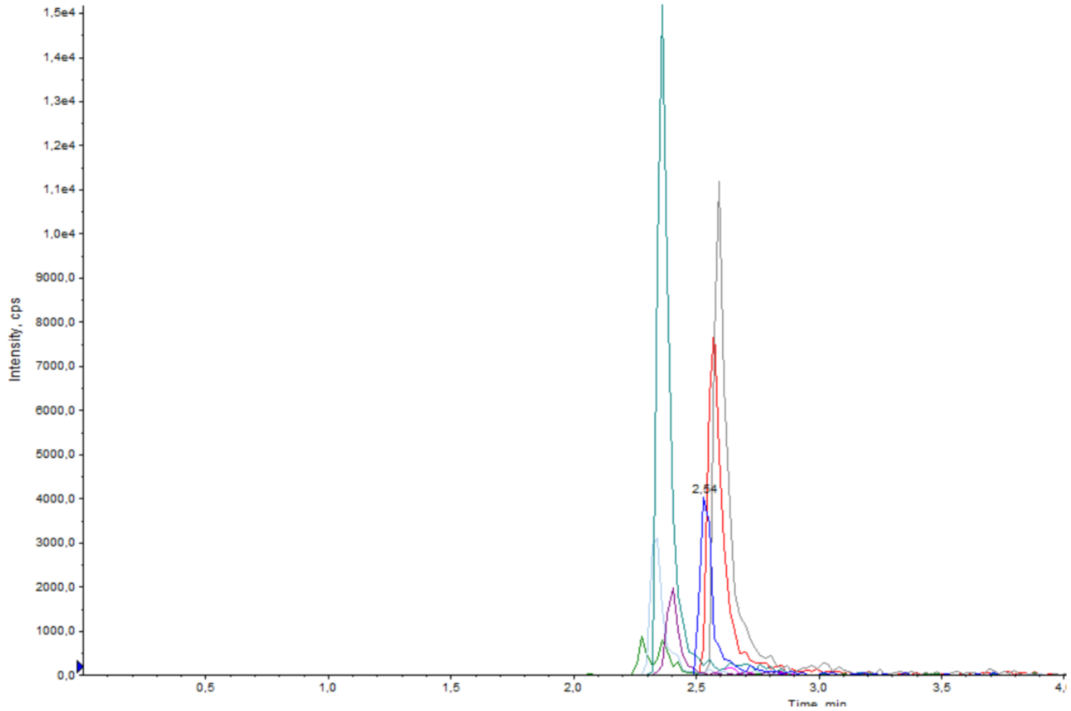

Figure S1. MRM chromatograms of surfactin (RT 2.52-2.59) and iturin (RT 2.29-2.41) **(a)** standards and **(b)** isolated from *Bacillus* sp. Kol L6.

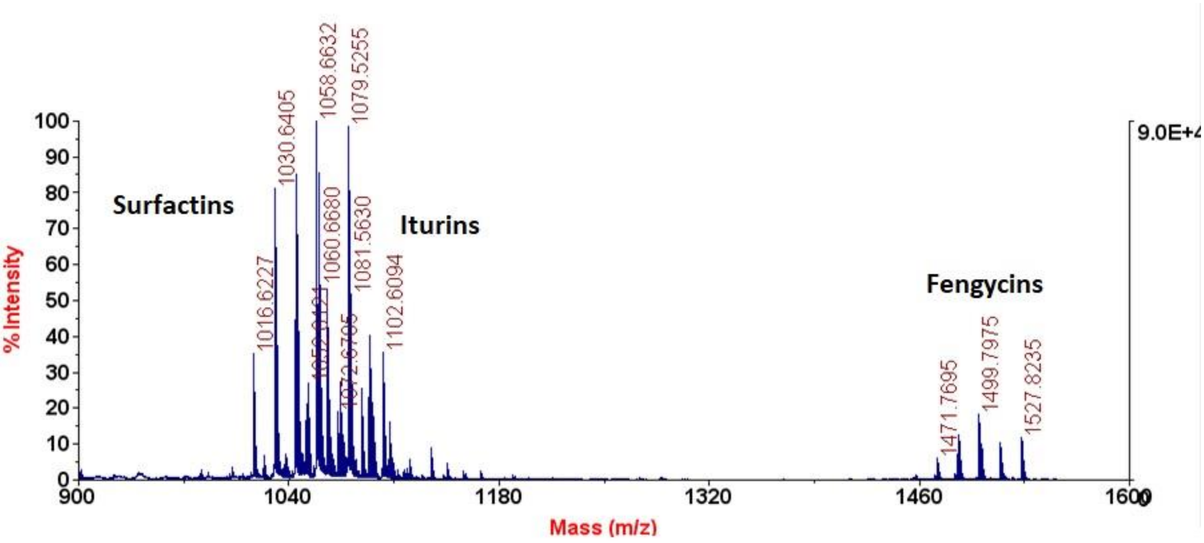

Figure S2. MALDI-TOF/TOF mass spectrum of the lipopeptide mixture presented in an extract prepared by a modified QuEChERS procedure from the culture supernatant of *Bacillus* sp. Kol L6.

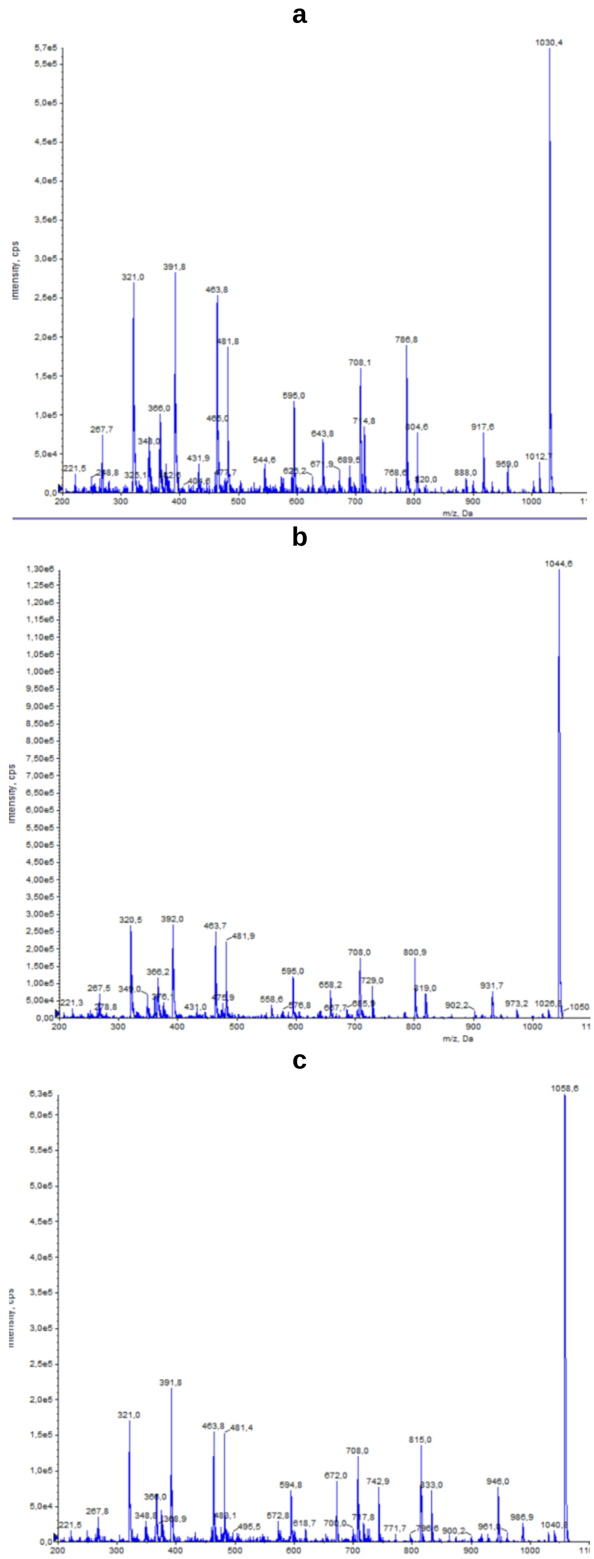

Figure S3. ESI-MS/MS spectra of surfactin homologues: **(a)** C13, **(b)** C14 and **(c)** C15.
